# Genes encoding novel secreted and transmembrane proteins are temporally and spatially regulated during *Drosophila melanogaster *embryogenesis

**DOI:** 10.1186/1741-7007-7-61

**Published:** 2009-09-22

**Authors:** Alejandro Zúñiga, Christian Hödar, Patricia Hanna, Freddy Ibáñez, Pablo Moreno, Rodrigo Pulgar, Luis Pastenes, Mauricio González, Verónica Cambiazo

**Affiliations:** 1 Laboratorio de Bioinformática y Expresión Génica, INTA-Universidad de Chile, Millennium Nucleus Center for Genomics of the Cell (CGC), Santiago, Chile

## Abstract

**Background:**

Morphogenetic events that shape the *Drosophila melanogaster *embryo are tightly controlled by a genetic program in which specific sets of genes are up-regulated. We used a suppressive subtractive hybridization procedure to identify a group of developmentally regulated genes during early stages of *D. melanogaster *embryogenesis. We studied the spatiotemporal activity of these genes in five different intervals covering 12 stages of embryogenesis.

**Results:**

Microarrays were constructed to confirm induction of expression and to determine the temporal profile of isolated subtracted cDNAs during embryo development. We identified a set of 118 genes whose expression levels increased significantly in at least one developmental interval compared with a reference interval. Of these genes, 53% had a phenotype and/or molecular function reported in the literature, whereas 47% were essentially uncharacterized. Clustering analysis revealed demarcated transcript groups with maximum gene activity at distinct developmental intervals. *In situ *hybridization assays were carried out on 23 uncharacterized genes, 15 of which proved to have spatiotemporally restricted expression patterns. Among these 15 uncharacterized genes, 13 were found to encode putative secreted and transmembrane proteins. For three of them we validated our protein sequence predictions by expressing their cDNAs in *Drosophila *S2R+ cells and analyzed the subcellular distribution of recombinant proteins. We then focused on the functional characterization of the gene CG6234. Inhibition of CG6234 by RNA interference resulted in morphological defects in embryos, suggesting the involvement of this gene in germ band retraction.

**Conclusion:**

Our data have yielded a list of developmentally regulated *D. melanogaster *genes and their expression profiles during embryogenesis and provide new information on the spatiotemporal expression patterns of several uncharacterized genes. In particular, we recovered a substantial number of unknown genes encoding putative secreted and transmembrane proteins, suggesting new components of signaling pathways that might be incorporated within the existing regulatory networks controlling *D. melanogaster *embryogenesis. These genes are also good candidates for additional targeted functional analyses similar to those we conducted for CG6234.

See related minireview by Vichas and Zallen:

## Background

Early stages of *Drosophila melanogaster *embryogenesis involve cellularization of the syncytial blastoderm and gastrulation. A series of morphogenetic events, which include cephalic and ventral furrow formation, posterior and anterior midgut invaginations, germ band extension and amnioserosa formation, initiate gastrulation and drive an extensive reorganization of the embryonic epithelium [1]. Concurrent with these processes, the expression of zygotic transcripts is activated for the first time at the stage that precedes gastrulation, when degradation of the maternal transcripts has already started [2]. At later stages of embryogenesis, additional morphogenetic events, germ band retraction, dorsal closure and head involution, shape the first instar larva [3]. The precise control of these cell and tissue rearrangements requires the integration of diverse molecular processes. First, transcriptional regulators assign positional cues and cell fate, thus specifying different cellular groups. Second, extracellular signals temporally and spatially coordinate the cellular behaviors that transform the embryo's epithelium. Finally, changes in the cellular architecture are supported by the activity of cytoskeletal regulators and cytoskeletal binding proteins [1,4]. Certain common molecular components, such as actin-myosin complexes, are part of the central mechanisms involved in early development in various organisms [5-8]. Thus, the molecular components that regulate cell shape and movement seem to be ubiquitous, but morphogenetic changes take place only in specific clusters of cells. Therefore, regulatory pathways should exist that differentially modulate cell behavior, leading to well-orchestrated cell shape changes and cell movements. In this regard, secreted and cell surface molecules, which are critical for intercellular communication, are expected to regulate many aspects of development.

Genetic analysis of development in *D. melanogaster *has proven to be a powerful approach for studying the mechanisms of early embryogenesis, and most of the genes known to be involved in key developmental signaling pathways have been identified through classical genetic screens. Genetic techniques have certain limitations, however, because genes with subtle loss-of-function phenotypes or pleiotropic roles are unlikely to be identified. Furthermore, classical genetic screens do not focus on specific molecular classes (for example, secreted versus intracellular gene products). Accordingly, other approaches have been developed that allow rapid and comprehensive identification of secreted and transmembrane gene products. These include library preparation from RNA enriched by microsomal fractionation [9] and RNA-mediated interference (RNAi) screens in a *Drosophila *cell line [10].

Recent research has identified a host of genes that control diverse aspects of *Drosophila *embryo development. cDNA arrays have greatly accelerated the discovery of differentially expressed genes and opened up a broad spectrum of research possibilities. This technology has been applied, among others, to uncover neural precursor genes [11], to determine temporal gene expression patterns for almost two-thirds of the *D. melanogaster *genome during its life cycle [12,13], and to discover new genes involved in muscle differentiation [14,15] and dorsoventral axis specification [16].

As these studies collectively build a network of genetic interactions, it is necessary that we begin to identify comprehensive sets of genes active at the different developmental stages, in order to increase our understanding of the molecular mechanisms that regulate morphogenesis throughout embryo development. With this goal in mind we applied a suppression subtractive hybridization (SSH) procedure to isolate genes that are expressed at the beginning of gastrulation. Recording the temporal and spatial expression profiles of these genes will allow us to add critical details to the current models of cellular behavior during *D. melanogaster *morphogenesis.

In our current work we identified a small number of genes with temporally and spatially restricted patterns of expression in the *D. melanogaster *embryo. In particular, we recovered a subset of uncharacterized genes encoding putative secreted and transmembrane proteins, and for three of them we expressed their cDNAs in *D. melanogaster *S2R+ cells and analyzed the subcellular localization of the recombinant proteins, confirming our sequence predictions. One of these genes, *CG6234*, with a restricted dorsal expression in the developing embryo, was then functionally tested by RNAi. Inhibition of *CG6234 *resulted in morphogenetic defects reminiscent of those exhibited by already characterized genes with roles in amnioserosa maintenance and/or differentiation. Our results contribute to the goal of finding all the genes involved in *D. melanogaster *embryogenesis by identifying a collection of genes that have not been previously implicated in development but have expression patterns suggestive of potential developmental roles.

## Results and discussion

### Subtracted cDNA library composition and characterization

To isolate transcripts that are differentially expressed at early stages of *Drosophila *development, we performed a SSH procedure between gastrulation and syncytial blastoderm stages, using cDNA prepared from stage 6-7 embryos (gastrula) as tester and that from stage 2-3 embryos (syncytial blastoderm) as driver, thus generating a cDNA population enriched in cDNA fragments that were expressed at a higher level in gastrula. As a control, SSH was also conducted reversely using the cDNA prepared from stage 2-3 and stage 6-7 embryos as tester and driver, respectively. To evaluate the effectiveness of subtraction, we performed a set of experiments (see Additional file 1A to 1C: Verification of SSH procedure) showing that the *actin *transcript was indeed over-represented five-fold in unsubtracted samples compared with the subtracted samples for both forward and reverse reactions (Additional file 1A). Thus, in our experimental conditions, subtractive hybridization removed common and housekeeping genes.

The pool of subtracted cDNA fragments ranged from approximately 300 bp to approximately 800 kb, with most of the fragments (>80%) distributed between 400 and 600 bp. These fragments were inserted into a T/A cloning vector, and the resultant SSH library was used for sequence analysis. In addition, a subset of clones was randomly selected to determine the efficiency of subtraction using a manually spotted cDNA microarray. The results indicated that 96 of 126 (76%) cDNA fragments tested displayed higher levels (>twofold) of expression in gastrula compared with syncytial blastoderm (Additional file 1B). To provide further data on relative expression levels of the cloned cDNAs, 10 positive clones were selected for virtual northern blot analysis; all cDNAs were up-regulated in gastrula (Additional file 1C), thus validating the performance of the protocol.

Of 1,440 subtracted clones, 642 were randomly selected for sequencing. The sequences with poor quality or shorter than 50 bp were eliminated from further analyses. The sequences reported in this paper have been deposited in GenBank: accession numbers FF579035-FF579613. By BLAST analysis against sequence databases available at FlyBase [17] we identified 254 non-redundant cDNAs that showed good matches (E-value < 1E^-10^) with sequences of the CDS (coding sequence) database, indicating that they correspond to protein-coding genes. In addition, four non-coding RNA (ncRNA), five transposition elements, and 39 sequences annotated as introns or as intergenic regions were identified. Among the sequences that mapped to intergenic and intronic regions, 17 of them had matches (E-value < 1E^-7^) with *D. melanogaster *ESTs at the Berkeley Drosophila Genome Project, suggesting that they might represent true transcripts. In this regard, using the ORESTES methodology, Maia et al. [18] identified 68 potentially transcribed regions derived from regions unannotated in the version 4.3 of the *D. melanogaster *genome. Experimental validation of unannotated ORESTES revealed 17 new exons of low-abundance transcripts. Thus, some *D. melanogaster *genes or gene variants may still remain to be discovered.

Regarding the 254 protein-coding genes isolated in this screen, we used the database of Gene Ontology [19], together with domain searches performed against public databases, to proceed with the functional classification of this set of subtracted genes (Additional file 2: Functional composition of the subtracted library). The GO annotations were contrasted with GO annotation of the entire *D. melanogaster *genome, and we found that the relative counts of subtracted genes were different from that of the *D. melanogaster *genome in the second-level GO categories (Additional file 2, compare red and blue bars). In the subtracted library the three major classes of GO 'molecular functions' were: binding (47.6%), transcription regulator activity (10.6%), and catalytic activity (23.5%). The first two were over-represented in the subtracted library when compared with the whole genome annotation, whereas catalytic activity was under-represented. For GO 'biological processes' the most highly represented classes were cellular process (27%), developmental process (19%), and multicellular organismal process (17%). The last two were over-represented in the library and, consistent with the developmental stage used as a tester during the subtraction procedure, these two classes include genes whose products are involved in morphogenesis, pattern specification, cellularization, and intracellular transport, among others.

### Temporal patterns of gene expression

To show how transcriptional activity of subtracted genes was modulated as a function of time during the first 12 stages of *D. melanogaster *development, we designed and used microarrays containing either the complete subtracted library (579 cDNAs) or 302 non-redundant cDNAs (including coding sequences, ncRNAs, transposons, introns and intergenic regions) spotted onto nylon membranes. This approach has been previously used for gene expression profiling [20,21]. The data discussed in this work have been deposited in NCBI-Gene Expression Omnibus [22], under GEO Series Accession No. GSE15000. We selected embryos at successive intervals and prepared SMART-cDNAs to hybridize on microarrays. We attempted to obtain homogeneous populations of embryos at each developmental interval to increase the temporal resolution of the expression profiles. To that end, we hand-selected embryos according to morphological criteria [3] at five intervals: stages 2-3, syncytial blastoderms (S2-3); stage 5, cellular blastoderm (S5); stages 6-7, gastrula (S6-7); and postgastrulation stages (S8-9 and S10-12). SMART-cDNA probes were evaluated for their temporal specificity using quantitative real-time PCR (qPCR) to amplify the transcripts of stage-specific genes (data not shown). After microarray hybridizations, the data files generated from the microarray images were processed to remove low-quality spots and normalized as described in Methods. The results of this analysis showed that 118 genes (114 protein-coding genes and four ncRNAs) and five non-coding sequences were differentially expressed (False Discovery Rate (FDR) <5%) between any developmental interval and the reference interval (S2-3) as determined by Significance Analysis of Microarrays [23]. All of these genes were up-regulated at least at one of the examined intervals (Additional file 3: Genes up-regulated during embryo development). Most of the remaining cDNAs corresponded to spots of low-intensity signal, suggesting that microarray hybridization failed to reach the sensitivity of the SSH procedure to detect low-abundance transcripts. In this regard, it has been shown that the sensitivity of microarray analysis is determined by the targets used to hybridize the membranes but not by the probes printed on them [24].

**Figure 1:**
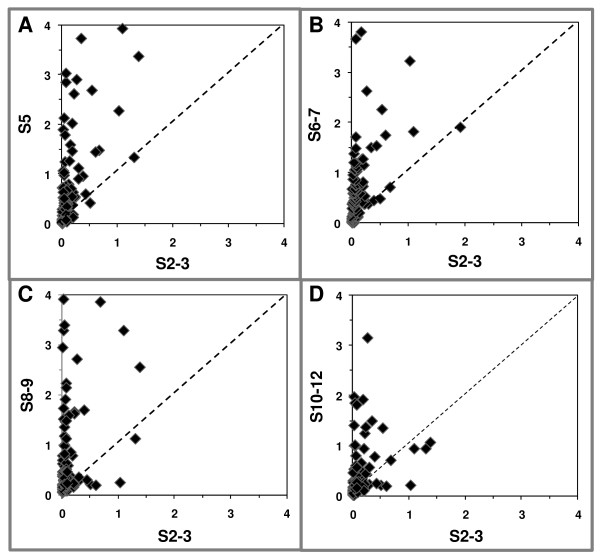
**Up-regulation of subtracted transcripts during development**. In each graph, the normalized hybridization signal intensities of genes whose expression changed significantly between any developmental interval and the reference interval were plotted on the *y *axis for stages 5 (S5; A), 6-7 (S6-7; B), 8-9 (S8-9; C) or 10-12 (S10-12; D), and on the *x *axis for stages 2-3 (S2-3). The diagonal line indicates no change in expression.

Signal intensity data resulting from genes that were differentially expressed in the microarray experiments were used to generate scatter plots, representing the ratio for each gene between a given developmental interval (*y *axis) and the reference S2-3 (Figure 1). Relative to S2-3, the highest frequency of up-regulated genes was observed at S5 and 6-7, and by S10-12 a major fraction of genes exhibited similar expression levels with S2-3 (represented by the diagonal line in Figure 1). Thus, the temporal expression levels of genes isolated in our screen indicate that their expression patterns change throughout the developmental intervals and that these shifts differed from gene to gene.

We compared the genes that were identified as up-regulated in this study with available data from reports on global gene expression analyses during *D. melanogaster *development (Additional file 3: Genes up-regulated during embryo development). Despite the differences in the procedure to obtain the cDNAs and in the hybridization techniques, we found that 27% of the up-regulated genes (*N *= 118) reported here were described as transiently expressed during early embryogenesis, as described [13], including genes previously characterized (*N *= 18) and unidentified genes (*N *= 15). Moreover, 17% of the genes that changed their expression levels during early stages of development belong to the group of early zygotic genes [12], whereas 8.5% of them (CG12420, CG4440, CG8960, *ptr*, *inx3*, *Kp78b*, CG13333, CG13427, *sep5*, CG6234) correspond to genes that were classified as specifically expressed during cellularization [25]. Finally, we found that 12% of the genes recovered from our microarray hybridization have been described as genes differentially expressed between gastrula and syncytial blastoderm stages [26].

When we analyzed the genes that significantly increased their expression levels in least at one interval compared with the S2-3, we distinguished the following classes: 63 genes have a phenotype or molecular function reported in the literature and have a name assigned; the remaining 55 genes either lack a name but have an assigned CG number, or have a name but are not functionally characterized. For 27 genes of these two last classes a GO term has been assigned on the basis of homology with known protein domains or genes in other species, whereas the remaining 28 genes are essentially uncharacterized. For some of them we were able to predict signal peptide sequences and/or transmembrane regions (Additional file 3: Genes up-regulated during embryo development).

**Figure 2:**
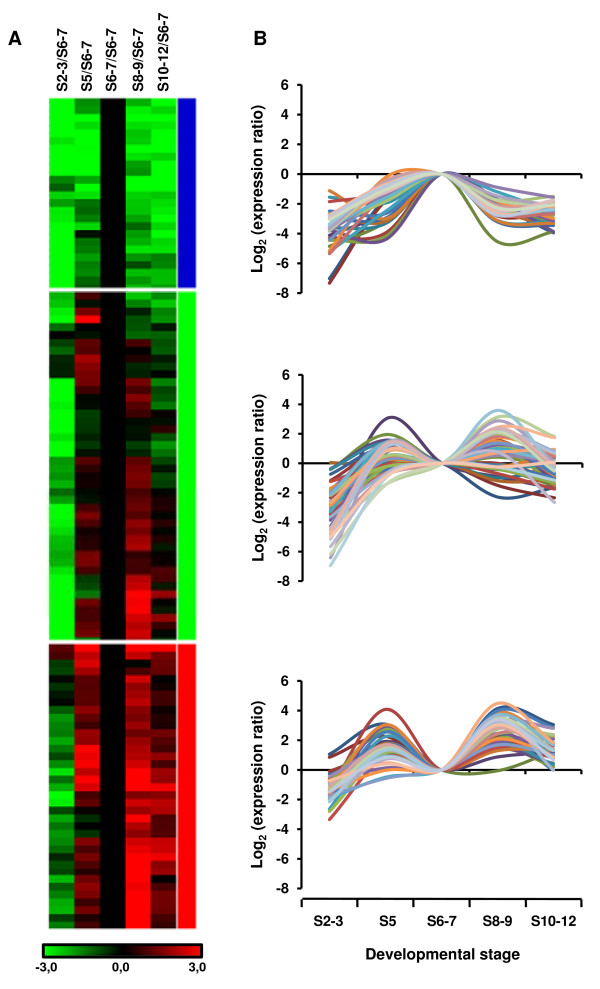
**Cluster analysis of microarray data**. (A) Hierarchical clustering was used to group the up-regulated genes (false discovery rate < 5%) based on similar expression patterns over the five developmental intervals examined (S2-3, S5, S6-7, S8-9 and S10-12 embryos, indicated above the columns). Gene expression patterns are evident across the rows. Increased and decreased expression compared with the mean expression of the S6-7 sample for each gene is shown in red and green, respectively. Green indicates log_2 _ratios < 0, and red indicates values >0. Three main gene groups were resolved and indicated by color bars shown to the right of the figure. (B) Graphs indicate genes that displayed a similar time-dependent expression pattern. *y *axis: log_2 _of expression ratio between each developmental interval and S6-7; *x *axis: developmental intervals.

Using data derived from the 114 protein-coding genes and four ncRNA genes, a clustering analysis was performed to group genes with similar expression profiles over the five developmental intervals (Figure 2). The analysis revealed three distinct patterns of gene expression along the developmental stages examined. The first group (blue bar) contains genes that show maximal activity at S6-7 and a rapid decrease in gene expression at later stages, indicating that our approach yielded a cDNA population enriched in transcripts corresponding to genes expressed during gastrulation. As an example, we analyzed gene composition in this cluster and found transcripts encoding proteins known to play roles during gastrulation, such as the transcriptional regulator *brk*, which is expressed in the neurogenic ectoderm and restricts the expression of Decapentaplegic (Dpp) target genes to the dorsal ectoderm [27], *tup*, a target of Dpp and member of the ush-group of genes required for amnioserosa maintenance [28], and *trn*, a transmembrane protein containing extracellular leucine-rich repeats [29]. The second and third groups (green and red bars, Figure 2) contain genes that showed a pattern of successive increase and decrease in gene expression, with peaks of expression at S5 and S8-9. In the second group (green bar), several of the genes showed a rapid decrease in expression at S10-12, whereas most of genes in group 3 (red bar) remained active at those late stages of embryogenesis.

The majority of the genes detected as up-regulated in our microarray analysis (69.5%) are contained within these two groups (red and green bars). They show peak expression during embryo cellularization, a transcriptional profile that seems consistent with the evidence that zygotic transcription starts at the beginning of cellular blastoderm formation [2] and with large-scale transcriptome analyses during *D. melanogaster *embryogenesis [12,13,25]. Among the genes with peak expression in S5, we recovered the *sry-α *gene, which encodes a protein essential for membrane invagination that is specifically required during cellularization [30]. Other genes showed more sustained expression at later stages of embryogenesis, for example: a) developmentally regulated transcription factors (*hth*, *Kr*, *h*, *ci*) with known expression patterns and roles in early embryonic development [31-34]; b) transmembrane proteins, such as *ptc*, the receptor of the morphogen Hedgehog [35,36] and *Nrt*, a cell adhesion molecule that initiates its expression during cellularization, and later on is restricted to neuronal precursors [37]; and c) components of intracellular signaling pathways, such as *stumps *(*dof*) that functions downstream of the fibroblast growth factor receptor during mesoderm migration and tracheal branching [38], and *vn*, a ligand of the epidermal growth factor receptor that participates in the patterning of the neuroectoderm during gastrulation [39].

On other hand, 38 genes (39%) have not been experimentally characterized in *D. melanogaster*; however, several of them encode products having sequence similarity to known proteins or containing conserved domains (Additional file 3: Genes up-regulated during embryo development). Among the genes encoding products with conserved domains, we found *patched-related *(*ptr*, CG11212), a transmembrane protein containing a sterol-sensing domain. We recently cloned the full-length cDNA of *ptr *and compared the amino acid sequence identity of the encoded protein among sterol-sensing domain-containing proteins from different species of insects and vertebrates, finding that Ptr belongs to a divergent, previously uncharacterized class of insect transmembrane protein. We also have demonstrated that *ptr *expression is developmentally regulated, being preferentially expressed in early embryo stages [40].

**Figure 3:**
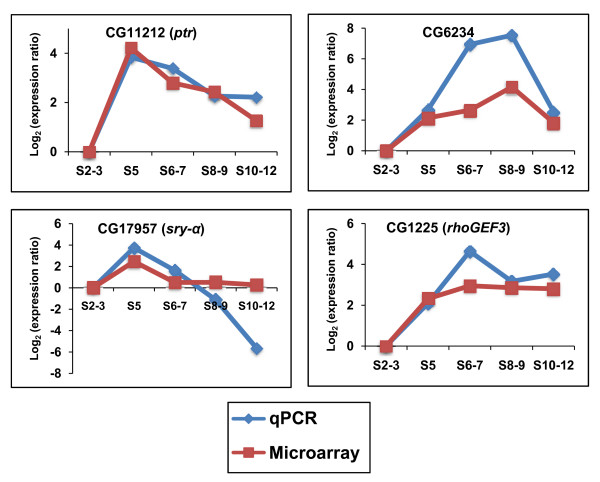
**Comparison between qPCR and microarray results for selected genes**. qPCR was done for four clones from Clusters 2 and 3 using template cDNAs obtained from stage 2-3 (S2-3), 5 (S5), 6-7 (6-7), 8-9 (S8-9) and 10-12 (S10-12) embryos. To calculate the ratios of expression for qPCR and microarray assays, data for S2-3 embryos were used as reference. The results are presented as the average of log_2 _ratios from replicate qPCR and microarray experiments.

To validate the gene expression profiles, qPCR analyses were performed for four genes (CG11212, CG6234, CG1225 and CG17957) at selected developmental stages, using independently isolated batches of total RNA extracted from staged embryos. Even though the quantities of transcripts detected could not be compared because of the different methods of estimation, the patterns of expression obtained via qPCR closely paralleled the microarray data (Figure 3).

### Spatial distribution of genes

To illustrate the spatial pattern of expression of genes differentially expressed in our microarray analysis, 28 genes were selected for *in situ *hybridization assays. Five of them corresponded to genes previously characterized (see below) with known patterns of expression, whereas the other 23 were selected among uncharacterized genes (Additional file 3: Genes up-regulated during embryo development). As 19 of 55 uncharacterized genes recovered from our microarray analysis encoded putative secreted or transmembrane proteins, we selected 15 of them to examine whether they displayed restricted patterns of expression in the developing embryo that might imply potential developmental roles. We chose to characterize genes encoding putative secreted or transmembrane proteins because they are expected to be active and play an important role in the temporal and spatial coordination of early *D. melanogaster *embryogenesis. In fact, secreted and membrane proteins have been reported to play a critical roles in *D. melanogaster *embryogenesis, as indicated by the functional characterization of genes involved in the Hedgehog [41], Dpp [42], and Wingless [43] signaling pathways, among others. Additional genes examined by *in situ *hybridization encoded products with conserved protein domains, such as DH-PH, metalloprotease, acetyltransferase, SH3, and phosphatase, and one mRNA-like ncRNA (Additional file 3: Genes up-regulated during embryo development).

**Figure 4:**
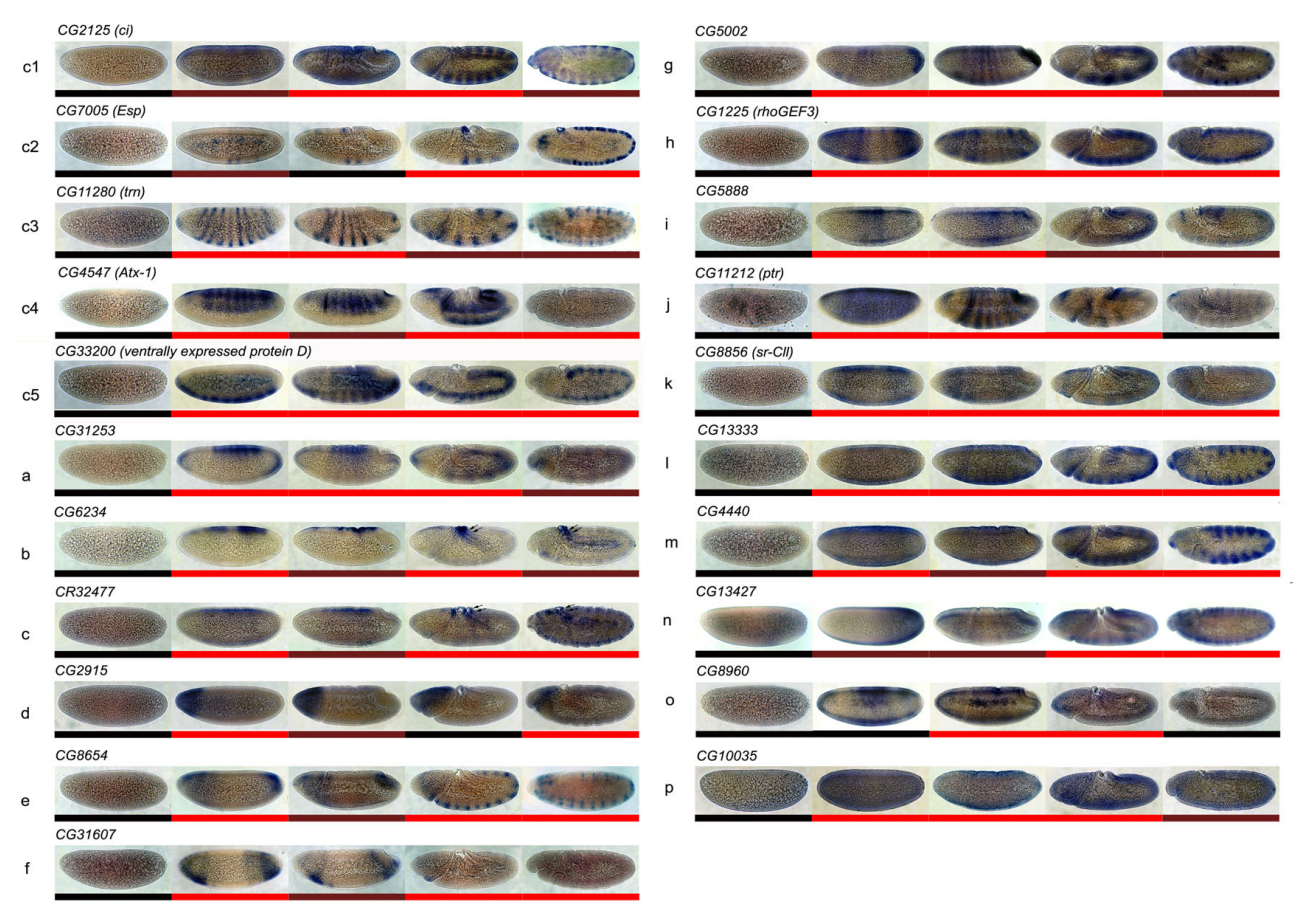
***In situ *hybridization of selected genes**. Representative images of whole-mount *in situ *hybridizations ordered according to developmental time to allow visual correlation with the corresponding microarray profile (from left to right: stages 2-3, 5, 6-7, 8-9, and 10-12). Embryos are oriented with the anterior region to the left and the dorsal region facing upward; all are lateral views. Gene symbols are indicated above the series of images. To compare the expression profile obtained by our microarray study and *in situ *hybridizations we transformed the gene expression values as follows: normalized intensity values (IV_i = stages 2-3, 5, 6-7, 8-9, 10-12_) of each gene at each developmental interval were used to build a gene expression vector (gev) with coordinates calculated as gev_i _= log_2 _(IV_i_/IV_S2-3_). Then, gev_i _arithmetic means (Mgev) for each gene were used to build a color scale according to the following rules: black, gev_i _= 0; dark red, 0 < gev_i _≤ 0.5 Mgev; red, gev_i _≥ 0.5 Mgev. The scale is represented by color bars at the bottom of each series of *in situ *images. Known genes, c1 to c5; uncharacterized genes, a to p.

Expression patterns obtained after *in situ *hybridization analysis were categorized as 'ubiquitous' (29%), if similar levels of expression were observed in all tissues, and as 'restricted' (71%), if transcripts were localized to just a few regions of the embryo in at least one of the stages examined. The expression patterns of all the restricted genes (20 of 28 tested genes) and one ubiquitous gene are illustrated in Figure 4; they correspond to 16 previously unidentified and five characterized genes: *ci *(CG2125; [34]), *ventrally expressed protein-D *(CG33200; [44]), *Esp *(CG7005; [45]) *trn *(CG11280; [29]) and *Atx-1 *(CG4547; [46]). In Figure 4, *in situ *images were ordered according to developmental time to allow visual correlation between microarray profiles and gene expression as revealed by the *in situ *assays. We categorized the expression changes (see Figure 4 legend) and represented each category with a color bar, using S2-3, with the lowest expression value (Mgev_i _= 0, black bar), as a reference. As expected, undetectable or very weak expression was observed for the entire set of genes at S2-3 (Figure 4). The five characterized genes reproduce the expression patterns described in the literature and showed good correlation between microarray and *in situ *image data (Figure 4 panels c1-c5). In the case of uncharacterized genes with restricted patterns of expression, we found that *in situ *images of gene expression were consistent with the microarray results (Figure 4, compare blue staining and color bars). For gene CG2915, however, we could not correlate microarray data with *in situ *images of its expression at S8-9 (panel d). If the expression level of CG2915 is low and it is expressed only in a subset of cells, as is shown in the *in situ *image, it is possible that the microarray results from the whole-animal experiment may not have been sensitive enough to detect gene expression changes, resulting in a disparity with the microarray expression profile. For seven of the uncharacterized genes the expression patterns described here confirmed the *in situ *data available at BDGP gene expression database [47].

A significant fraction of the uncharacterized genes included in this assay exhibited spatially restricted patterns of expression during embryogenesis, including 12 of the 15 genes that encode putative secreted or transmembrane proteins, suggesting that they might play roles in development. In the cellular blastoderm and gastrula stages, several transcripts were asymmetrically distributed along the dorsoventral (Figure 4, panels a to c) or anteroposterior (Figures 4, panels e to i) axes. Other transcripts showed a more uniform distribution at the cellular blastoderm stage; some of them refined their expression pattern during gastrulation (CG11212, panel j, Figure 4) or after germ band extension (panels k to n, Figure 4), one of them became undetectable at later stages of embryogenesis (panel o, Figure 4), and one remained ubiquitous (panel p, Figure 4).

Two genes (CG6234 and CR32477) showed highly restricted distributions at the cellular blastoderm stage and a similar pattern of expression along the developmental stages examined (compare panels b and c in Figure 4), suggesting a shared gene regulation and/or functional relationship. Their transcripts were first detected at S5 in the dorsal-most cells of the embryo, and this expression pattern persisted during gastrulation to become enriched in the cells of the amnioserosa at later stages of embryogenesis (black arrows in panels b and c, Figure 4). Thus, the expression pattern of CG6234 and CR32477 suggests that these genes might be involved in the early events of amnioserosa formation. Consistent with the asymmetrical distribution of CG6234 transcripts along the dorsoventral axis, CG6234 seems to be an ectodermic transcriptional target of the Dorsal morphogen [48]. This gene encodes a protein that lacks any conserved domains or significant sequence similarity in the databases, thus precluding making inferences about its function(s) or interaction with other proteins. However, the CG6234 deduced amino acid sequence includes a predicted signal peptide at its amino terminus and a predicted transmembrane domain, suggesting that it is associated with membranes [26]. CR32477, on the other hand, is an uncharacterized putative mRNA-like ncRNA encoding several short open reading frames. In this regard, *in situ *hybridization analyses of 35 mRNA-like ncRNAs, whose transcripts are expressed during embryogenesis, revealed that 27 of them were detected in specific embryonic tissues [49]. Thus, restrictive spatial expression patterns might be a common feature of mRNA-like ncRNAs. Moreover, these highly regulated expression patterns suggest that many mRNA-like ncRNAs might play important roles in *D. melanogaster *embryogenesis, as previously demonstrated [50].

An additional gene that showed restricted patterns of expression at the cellular blastoderm stage encodes a member of the sulfate transporter family (CG5002, panel g, Figure 4). Interestingly, another gene encoding a sulfate transporter (*Esp*, panel c2, Figure 4) was also recovered from our microarray analysis; both of them are homologs of the human diastrophic dysplasia sulfate transporter (DTDST). Mutations in *DTDST *result in reduced sulfate transport, which in turn leads to defects in glycosaminoglycan (GAG) synthesis, under-sulfation of proteoglycans, and abnormal cartilage formation. To our knowledge, sulfate transporters have not been characterized in *D. melanogaster*; the temporal and spatial regulation of these two genes, however, suggests that they might play a role in GAG synthesis early during embryogenesis. In this respect, an analysis of *D. melanogaster *mutants with defects in sulfotransferases revealed the significance of sulfation of GAGs on growth factor signaling during development; for example, a mutation in the gene *sfl *causes defects in Wingless [51] and fibroblast growth factor signaling [52].

Another gene that seems to be a good candidate to play roles during embryogenesis is *RhoGEF3 *(panel h, Figure 4), which encodes a putative member of guanine nucleotide exchange factor (GEF) family of proteins [53]. The expression pattern of *RhoGEF3 *differs from that of the known *D. melanogaster *GEFs, because they are usually post-translationally regulated and maternally supplied, and their spatial distributions are ubiquitous during early development [6,54-56]. Thus, the restricted expression pattern of *Rhogef3 *(Figure 4, panel h) makes it one of the first examples of a GEF-encoding gene that is regulated at the transcriptional level during *D. melanogaster *development, suggesting that it might be required for the spatial and temporal control of actin dynamics. Five genes that showed restricted patterns of expression at the cellular blastoderm stage encoded putative secreted or transmembrane proteins; four of them, CG31253 (panel a), CG8654 (panel e), CG31607 (panel f) and CG5888 (panel i) in Figure 4, lack any other conserved domain, whereas CG2915 (panel d) seems to encode a putative secreted metalloprotease that showed restricted expression in the procephalic embryo region, making it a good candidate for targeted functional studies.

Among the transcripts that had restricted localization after the cellular blastoderm stage, we found CG11212 (*ptr*, panel j, Figure 4 which encodes a transmembrane protein containing a sterol-sensing domain [40], CG8856 (panel k, Figure 4), encoding a putative scavenger receptor [57], and two genes CG13333 and CG4440 (panels l and m, Figure 4), encoding putative secreted proteins. CG13333 and CG4440 were recently identified [13] as genes transiently expressed during embryo development. As the temporal expression pattern of CG13333 and CG4440 correlated well with the expression of several members of the Notch pathway, including Notch itself, the authors examined whether these two genes colocalized with the Notch pathway by using Delta as a marker. The results of their spatial colocalization allowed them to suggest that CG1333 and CG4440 are implicated in Notch-regulated developmental processes. Finally, the potential roles of genes CG13427 (panel n, Figure 4), CG8960 (panel o, Figure 4) and CG10035 (panel p, Figure 4) were more elusive; they encode putative secreted or transmembrane proteins, but no further inferences about their biochemical activities could be made from their protein sequence. Nevertheless, their temporal expression patterns during *D. melanogaster *embryogenesis have been consistently reported by others [12,13,25,26,58].

Taken together, our analysis of gene expression during *D. melanogaster *embryogenesis not only confirmed a number of known expression patterns but also revealed several developmentally restricted uncharacterized genes with common temporal expression patterns that should be the target of functional studies and become integrated into the developmental networks that are active during early embryogenesis in *D. melanogaster*. Thus, our results complement previous high-throughput screens in *D. melanogaster *embryos [58,59], which revealed that a significant fraction of uncharacterized transcripts exhibited spatially restricted patterns of expression in the developing embryo, suggesting that they play distinct roles during embryogenesis.

### Characterization of new genes encoding putative secreted and transmembrane proteins

**Figure 5:**
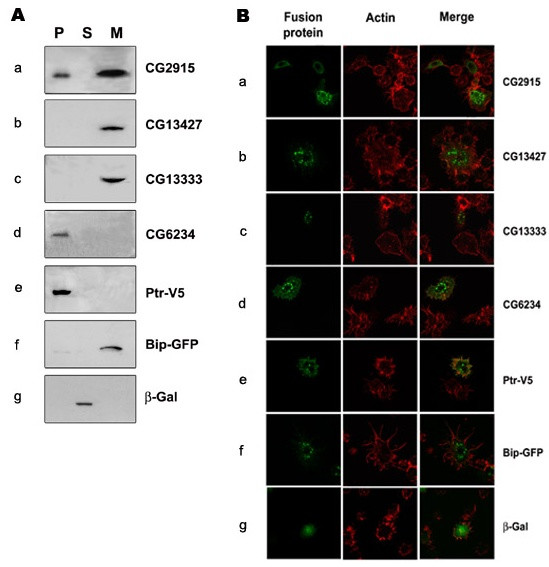
**Subcellular localization of uncharacterized proteins**. S2R+ cells were transiently transfected with plasmids expressing tagged CG2915, CG13427 CG1333 and CG6234 recombinant proteins. As controls for secreted, cytoplasmic, and transmembrane proteins, cells were transfected with constructs expressing V5-tagged Bip-GFP, β-Gal and Ptr fusion proteins, respectively. (A) Transfected cells were fractionated to obtain high-speed pellets (P) and supernatants (S). Equivalent volumes of cell fractions plus the medium in which cells were grown (M) were loaded on the gel and subjected to western blot analysis with anti-V5 (panels a and b and d to g) or anti-myc (panel c). (B) Transfected S2R+ cells were stained with anti-V5 (green in a and b and d to g) or anti-myc (green in c) and phalloidin to visualize actin (red in panels a to g).

We attempted to further characterize four of the uncharacterized genes identified in our screen that encode putative secreted and transmembrane proteins, CG13427, CG13333, CG2915 and CG6234. To confirm that these proteins are secreted or associated with membranes we transiently transfected *Drosophila *S2R+ cells with plasmid constructs encoding the cDNA of each protein fused to a V5 tag or a Myc tag sequence under the control of an inducible metallothionein promoter. After induction with CuSO_4_, we recovered the culture medium in which transfected cells had grown and cells were disrupted and centrifuged (see Methods) to obtain post-nuclear supernatants and corresponding pellets. Cell fractions and culture medium were examined by western blotting. As controls for the expected distributions for secreted, cytoplasmic, and transmembrane proteins, we performed parallel transfections with vectors expressing the signal sequence of *Drosophila *Bip protein appended to green fluorescent protein (GFP) and to V5 tag (Bip-GFP), the cytoplasmic protein β-galactosidase fused to V5 tag (β-Gal), and the *Drosophila *transmembrane protein Ptr fused to V5 (Ptr-V5 in Figure 5A). As shown in Figure 5A, CG13427, CG13333 and CG2915 fusion proteins were predominantly detected in the culture medium (Figure 5A, lane M), confirming that they are secreted proteins. In the case of CG6234, the fusion protein was detected exclusively in the pellet fraction, indicating that it is associated with particulate components of cells, including cellular membranes (Figure 5A, lane P). No fusion protein was found in the supernatant fraction (Figure 5A, lane S), except for the β-galactosidase control that lacks a signal peptide and transmembrane domain. These results confirm our prediction of four putative secreted or transmembrane proteins encoded by previously uncharacterized *D. melanogaster *genes.

The subcellular localization of the fusion proteins was examined by double immunofluorescence experiments using an antibody that recognizes the protein tags and Alexa 546-phalloidin to label actin filaments (Figure 5B). Controls of uninduced cells showed no reactivity with anti-V5 or anti-myc (data not shown). These experiments showed that transfected S2R+ cells stained positively for CG13427, CG13333, CG2915 and CG6234 fusion proteins in vesicle-like structures (Figure 5B). In addition, a fine granular fluorescence was observed all over the surface of cells expressing CG6234-V5, consistent with the detection of the tagged protein in the plasma membrane fraction. Thus, subcellular localization along with the restricted temporal and spatial patterns of expression that these genes displayed during *D. melanogaster *embryogenesis make them good candidates for functional studies.

As a first step towards functional analysis, we selected gene CG6234 for further study, using heritable RNAi technology to reduce its expression. We chose this experimental approach because no CG6234 mutant or suitable P-element insertion (that could be used to generate CG6234 mutants) exists. Thus, we generated a transgenic line that expresses an inverted repeat of a 560-bp region of the CG6234 coding sequence under the control of the UAS promoter in the vector pWIZ (UAS-*CG6234IR*). The transgenic flies were crossed with flies carrying the *nanos*-GAL4 driver to activate transcription of the hairpin-encoding transgene in the progeny. As a control, UAS-*CG6234IR *and GAL4 drivers were crossed with *w*^1118 ^flies. We examined by qPCR the CG6234 transcription level in F1 embryos and found that the amount of CG6234 mRNA in *nanos*-GAL4/UAS-*CG6234IR *embryos was 30 to 35% of the control embryos (data not shown).

**Figure 6:**
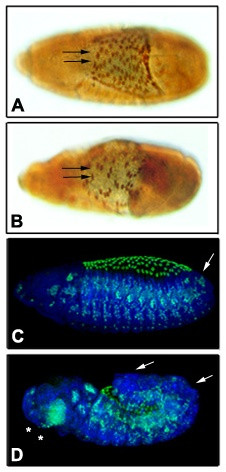
**UAS-*CG6234IR *induction in *D. melanogaster *embryos**. (A and B) Whole-mount immunohistochemical images of embryos (dorsal views) stained with anti-Hnt. (A) A wild-type embryo (stage 12) during germ band retraction. (B) An embryo expressing UAS-*CG6234IR *induced by *nanos*-GAL4. Hnt-positive nuclei of amnioserosa cells are indicated by black arrows. (C and D) Immunofluorescence confocal images of embryos (lateral views) stained with anti-Hnt (green) and To-PRO3 (blue). (C) Wild-type (stage 13) showing the amnioserosa nuclei that cover the dorsal region as a result of germ band retraction (white arrow). (D) UAS-*CG6234IR *ubiquitously expressed under *nanos*-GAL4 control produces embryos having disrupted germ band retraction (white arrows) and head defects (asterisks).

As CG6234 is mainly expressed in the primordial and developing amnioserosa, we used the amnioserosa marker Hindsight (Hnt) [60] to examine the phenotype of embryos expressing UAS-*CG6234IR*. F1 embryos showed a reduced number of Hnt-positive nuclei in the amnioserosa (Figure 6A and 6B, black arrows), suggesting the loss of amnioserosa cells. Two morphogenetic processes are known to require proper amnioserosa integrity: these are germ band retraction, which transforms the u-shaped germ band of the embryo by bringing its tail end to the final posterior position, and dorsal closure, a process that follows germ band retraction and seals the epidermis along the dorsal midline (see [3] for description). Consistent with a loss of amnioserosa integrity, embryos expressing UAS-*CG6234IR *displayed morphological defects; of note, the process of germ band retraction was severely disrupted (Figure 6C and 6D, white arrows). In addition, the embryos showed head defects, suggesting a failure to undergo head involution (Figure 6C and 6D, asterisks), a developmental process that leads to internalization of anterior ectodermal tissue and that seems to share genetic components with dorsal closure (reviewed in [61]). Thus, our results indicate that inducible down-regulation of CG6234 expression affects major morphogenetic events during embryo development. Moreover, the phenotype of UAS-*CG6234IR *is shared with previously described genes, among them genes that control amnioserosa cell death, such as *hnt*, *tup*, *ush*, *srp *and *doc *[28,62]. These genes are predominantly expressed in the amnioserosa, and when mutated they disrupt germ band retraction and in some cases they also affect head involution. In the case of CG6234, further functional studies will be necessary to place it within the known pathways that regulate these morphogenetic events.

## Conclusion

To understand the dynamics of the gene networks that underlie morphogenetic events of *D. melanogaster *embryogenesis, it is necessary to identify and characterize genes that are active at different developmental stages. The screen we described here allowed us to isolate genes differentially expressed between the gastrula and syncytial blastoderm stages. Microarrays were constructed to analyze the temporal expression patterns of subtracted genes during five developmental intervals that covered 12 stages of *D. melanogaster *embryogenesis. The results indicate that the expression of 118 genes, including 55 functionally unknown genes, increased significantly at least at one developmental interval compared with reference stages (S2-3). The large number of functionally uncharacterized genes (47%) identified as up-regulated during the developmental stages examined indicate that the SSH procedure together with the precise selection of staged embryos may be useful in providing information on uncharacterized genes having potential function at specific developmental stages.

In the same context, our results on the spatial distribution of 28 up-regulated genes indicate that a significant fraction of them (71%) exhibited restricted expression patterns during embryonic development. Their transcripts were detected in a variety of temporal and spatial expression domains, suggesting distinct roles during embryogenesis and making them good candidates for functional analyses. In addition, protein sequence analysis and motif identification revealed a set of uncharacterized gene products with putative functions as secreted or transmembrane proteins. For some of them, we validated our sequence predictions by analyzing their subcellular localization, thus providing new insights into effector molecules that might function in intercellular communication during *D. melanogaster *embryogenesis.

Using RNAi, we explored the function of one uncharacterized gene, CG6234, which showed a dorsally restricted expression pattern during embryo development. Phenotypic alterations of embryos expressing CG6234-RNAi support the idea that CG6234 might play a role in embryo morphogenesis. The CG6234 RNAi phenotype was reminiscent of that exhibited by previously characterized genes that function in amnioserosa maintenance and/or differentiation. Interestingly, they shared similar temporal and spatial expression patterns with CG6234, suggesting a common mechanism of transcriptional regulation. Thus our results provide information on new components of signaling pathways that might be incorporated within the known networks regulating *D. melanogaster *embryogenesis.

Finally, we note that even though *D. melanogaster *embryogenesis may appear to be a special case of morphogenesis, the cellular phenomena and the molecules involved are clearly general. Moreover, comparison of gene expression patterns and gene functions at key points during development reveals several molecular pathways that are common to bilaterian embryos. As *D. melanogaster *is the model organism that has provided much of our knowledge of embryogenesis and developmental genetics, it is relevant to understand more deeply the molecular processes underlying morphogenetic events in this organism and to discover and characterize the effector molecules that make effective cell-shape changes and promote cell migration.

## Methods

### Fly culture and embryo selection

Fly stocks were grown at 22°C on standard cornmeal, molasses, agar and yeast medium. Embryos were collected and hand-selected as described in Gonzalez-Agüero et al. [26]. *w*^1118 ^and the driver P{GAL4-nos.NGT}40 (*nanos*-GAL4) with a ubiquitous expression of GAL4 were obtained from the Bloomington *Drosophila *Stock Center.

### RNA extraction and cDNA synthesis

Total RNA was extracted from staged embryos (*N *= 100 to 150) using the RNA_WIZ _reagent (Ambion, Austin, TX, USA). Embryos were carefully homogenized in a 1.5 mL Eppendorf tube with 1 mL of RNA_WIZ _reagent using a plastic tissue grinder. To improve RNA yield, the homogenate was passed throughout Qiashredder columns (Qiagen, Venlo, The Netherlands) by centrifugation at room temperature for 2 min at 10,000 × *g*. RNA quantity and quality were assessed by OD_260/280 _and by electrophoresis on a 1.2% formaldehyde-agarose gel. Typical yield was 0.18 to 0.23 μg RNA/embryo. For subtractive hybridization procedure and microarray probe preparation, 0.5 μg of total embryo RNA was used to produce double-strand (ds) cDNA using the SMART™ PCR cDNA Synthesis kit (BD Clontech, San Jose, CA, USA) with 17 cycles of amplification. For qPCR, 1 μg of total RNA was used as a template for reverse transcription reactions to synthesize single strand (ss) cDNA using MMLV-RT reverse transcriptase (Promega, Madison, USA) and oligo-dT primer (Invitrogen, Carlsbad, CA, USA), according to standard procedures. A poly(A)-RNA was *in vitro *transcribed from the vector pGIBS-*dap *(ATCC 87486) and added to the embryo RNA samples prior to cDNA synthesis in a 1/1000 ratio, to be used as spike mRNA [63].

### Subtractive hybridization and library construction

An SSH procedure [64] was performed using driver dscDNAs from stage 6-7 embryos (gastrula) and tester dscDNA from stage 2-3 embryos (syncytial blastoderm). We performed the cDNA subtraction using the PCR-select cDNA subtraction kit (BD Clontech) according to manufacturer's recommendations with the following modifications: we used a driver/tester ratio of 2:1 (v:v) in the first hybridization and carried out 25 cycles of primary PCR and 12 cycles of secondary PCR with the Advantage cDNA polymerase mix (BD Clontech). To evaluate the efficiency of the cDNA subtraction, we compared the transcript levels of the housekeeping gene *actin *by qPCR (see below). Furthermore, to assess the efficiency of enrichment of differentially expressed genes, the abundance of the transcription factor *twist *was examined. As expected, transcripts of *twist *were enriched in the gastrula-subtracted sample as compared with the unsubtracted sample. The *twist*-specific product was detectable after 20 cycles of conventional PCR amplification when subtraction had been carried out, but not until 25 cycles in the corresponding unsubtracted sample. As described by BD Clontech, we estimate at least a 20-fold enrichment in the subtracted cDNA population data not shown.

The subtracted cDNA library was made from 100 μL of secondary PCR reaction from gastrula-subtracted cDNA sequences. PCR products were purified using the Wizard DNA Clean-up system (Promega), and 1 μL was inserted into the T/A cloning vector pGEM-T Easy (Promega) following the manufacturer's recommendations. A total of 1,440 individual transformants carrying cDNA fragments were isolated from white colonies on X-gal/IPTG agar plates, and individual clones were placed in an arrayed 96-well format.

To estimate the size of the inserts, plasmid DNA preparations were made from 126 cDNA clones by alkaline lyses [61], digested with *Eco*RI, and analyzed by electrophoresis on a 1.2% agarose gel. To evaluate whether the cDNA library was enriched in gastrula transcripts the inserts of the 126 cDNA clones were PCR amplified, manually spotted onto a nylon membrane, and hybridized with ^32^P-labeled cDNA probes synthesized from gastrula and syncytial blastoderm embryos as described [26]. Hybridization spots were visualized by exposure to X-ray films at -80°C for 6 h to 2 days. The intensity of the spots was measured with the Kodak 1D, v.3.5 software. The average intensity of the spots for each clone minus the average of total background intensity was normalized using the values obtained for the *actin *gene (Additional file 1B).

To perform virtual northern blot assays, SMART-cDNA (2 μg) from syncytial blastoderm and gastrula embryos were fractionated on 1.0% TBE-agarose gels for 3 h at 100 mV. Samples were transferred onto a positively charged nylon membrane using the alkaline capillary method [65] and then cross-linked by UV irradiation. DNA inserts from 10 clones were labeled with [^32^P]dCTP using the Random Primer Labeling Method (Invitrogen). Membranes were re-probed with ^32^P-labeled actin probe. Membrane prehybridization, hybridization and washing were as described [26]. Automatic sequencing of 642 clones at the 5' end was carried out by Agencourt Bioscience Corporation (Beverly, MA, USA). Sequence chromatograms were read with PHRED base calling software and filtered to improve the quality of sequence assembly. Clones containing sequences shorter than 50 bp were eliminated from further analyses.

### Bioinformatics

Sequence homology searches were performed using the standalone BLAST suite against *D. melanogaster *sequence databases (release 5.4) available in the FlyBase repository [66]. Unique hits were assigned when identity and E-values were >70% and <10E^-3^, respectively. BLAST analysis of intergenic and intronic sequences was performed against the EST database available at the Berkeley Drosophila Genome Project [67]. Domain-based analyses used SMART [68] and Interpro [69]. Signal peptide prediction used SignalP [70]. Transmembrane helix prediction used TMHMM [71]. Gene ontology annotation for every CDS entry (FlyBase release 5.4) was obtained by batch downloading from the FlyBase site [72]. Three branches of ontology were considered (molecular function, biological process and cellular component). From this set, the GO annotations assigned to the genes of the subtracted library were obtained. A Perl script relying on Perl package GO-TermFinder [73] was written to obtain the GO path for every annotated CDS. For that, GO format text files for the three different branches were used, they were obtained from the Gene Ontology site [74]. A CDS can have none, one or more GO numbers assigned per branch. Each GO number can have one or more paths to the base number of the acyclic graph. With the path of every assigned GO number, all the child terms of each GO number in the second level of each branch were counted. Counts were separately performed for the subtracted library hits and for the list of all CDSs in the genome.

### Microarray production

Clones were picked from -80°C stocks and grown overnight in 96-well plates in 200 μL LB containing 50 μg/mL ampicillin. PCR amplification of bacterial cultures and quality verification of the products were performed as described [75]. PicoGreen dscDNA Quantitation kit (Molecular Probes, Eugene, OR, USA) was used according to the manufacturer's instructions to quantify and standardize PCR product concentrations. PCR products (20 μL) were arrayed in 96-well plates and mixed with an equal volume of dimethyl sulfoxide (DMSO). PCR products (70 ng) were spotted in duplicate on 8 × 12 cm nylon membranes (GeneScreen Plus, Bio-Rad, Hercules, CA, USA) using an 8-pin print head (Arraylt model SSP015) and the arraying robot VersArray Chip Writer Compact (Bio-Rad). The membranes were treated as described [75]. In addition to the library clones, the following controls were spotted onto membranes: (1) a fragment of the vector pBluescript II obtained by amplification with the T7 and SP6 universal primers; (2) several spots of 50% (v/v) DMSO; (3) PCR-amplified fragments of genes *serendipity α*, *twist*, *tinman*, *fog *and *snail *as positive controls; (4) *actin*, *tubulin*, and *RP49 *as housekeeping genes, and (5) four dilutions of a PCR-amplified fragment from a *Bacillus subtilis dap *cDNA (ATCC; number 87486).

### Probe synthesis and membrane hybridization

The ^32^P-labeled probes from stage 2-3, 5, 6-7, 8-9 and 10-12 embryos were prepared from SMART-cDNAs by incorporation of [α-^32^P]dCTP using the Random Primers DNA Labeling System (Invitrogen) according to the manufacturer's instructions. Unincorporated radioactive nucleotides were removed using the QIAquick Nucleotide Removal kit (Qiagen). The labeled cDNA products were denatured and immediately used for membrane hybridization as described [75]. Membranes were sealed in plastic bags and placed in an Imaging Screen-K (Bio-Rad) for 12 to 24 h.

### Microarray experimental design and data analysis

Microarray experiments were performed in two independent labeling/hybridization events of cDNA probes. Radioactive images of the 20 hybridized membranes (out of 45) were obtained using a scanner Personal Molecular Image FX (Bio-Rad) at 50 μm/pixel resolution. Intensity values were measured using VersArray Analyzer v.4.5.1.46 (Bio-Rad). Local background values were measured in the corners of spots and were subtracted from the signal intensity values for each spot. Pearson's correlation coefficient was used to establish the quality of replicated membranes. Spots that showed: (1) Signal Mean < (Background Mean + (1 × Background Standard Deviation), (2) coefficient of variation > 0.5 between duplicate spots within membranes [76], or (3) qcom < 0.8 [77] were considered as low-quality spots and were removed. After data filtering, net intensity values were normalized against the housekeeping gene *RP49 *and *dap *spike mRNA intensity values. When genes were represented by more than one clone, mean values were calculated. To detect genes (and non-coding sequences) differentially expressed between stages 2-3 and any other developmental interval (in all the arrays tested), we performed a Significance Analysis of Microarrays [23]. A predicted FDR of 0.05 was used as the threshold for differential expression. Genes for which expression level changed significantly (that is, by at least at one time interval) were subjected to a hierarchical cluster analysis [78], using MeV v4.0 software [79], with average distances as parameter and the Pearson correlation as verification criteria.

### Quantitative real-time PCR (qPCR)

qPCR amplifications and fluorescence detection were performed using the LightCycler^® ^1.5 Instrument (Roche, Basel, Switzerland) and LightCycler^® ^FastStart DNA Master SYBR^® ^Green I (Roche). Reactions contained 100 ng of dscDNA or 50 ng of sscDNA. Primers were designed using Primer Premier 5.0 software (Palo Alto, CA, USA) and synthesized by Alpha DNA, (Montreal, Quebec). Primer sequences, annealing temperatures and amplicon lengths are given in Additional file 4 (Primers used for qPCR and conventional PCR reactions). For each gene, a calibration curve was generated based on serial dilutions (10^1 ^to 10^2 ^pg/μL) of plasmid templates. The thermal cycle conditions were: denaturation at 95°C for 10 min, followed by 35 three-step cycles of template denaturation at 95°C with a 2 s hold, primer annealing at 60 to 65°C for 15 s, and extension at 72°C for 60 s/1000 bp. The purity of amplified products was verified by melting curve analyses. Control reactions included a subset of PCR components lacking the cDNA template. The initial amount of transcript in each sample was calculated from the standard curve using the default (fit point/arithmetic) method of LightCycler Software Version 3.5, and normalized to the values of *actin *or *dap*. Data represent the mean of three experimental replicates.

### *In situ *hybridization of whole-mount embryos

*In situ *hybridization using 0.5 to 2 ng/μL DIG-labeled RNA probes was carried out as described [80], with the following modifications. After embryo collection, methanol-washed embryos were re-fixed for 20 min in post-fix solution (5% formaldehyde in phosphate-buffered saline (PBS), 0.1% Tween), and rinsed with PBS, 0.1% Tween (PBT). The embryos were treated with 3.5 μL of proteinase K (Roche, 50 μg/mL) in 1 mL of PBT for 3 min. Embryos were incubated with alkaline phosphatase-conjugated anti-digoxigenin antibody (Boehringer Mannheim; 1:2000) at room temperature for 3 h. The embryos were then washed extensively with PBT, and expression patterns were visualized by incubating them with staining solution containing NBT and BCIP (Vector Labs, Burlingame, CA, USA) as substrates. The reaction was stopped by washing the samples with PBT containing 20 mM EDTA. Stained embryos were dehydrated in a series of ethanol and xylene, mounted in Cytoseal TMXYL (Richard-Allan Scientific, Kalamazoo, MI, USA), and photographed on a Zeiss Axiovert 25 microscope with a Sony CyberShot Camera model DSC-S75 equipped with an Adapter Ring Vad-S70. Image files were processed using Adobe PhotoShop 7.0.

### Transfection of S2R+ cells

Specific primers were used to amplify the coding sequences of genes CG2915 (encoding amino acid residues 1 to 206), CG13427 (residues 1 to 104), CG6234 (residues 1 to 559), CG11212 (residues 1 to 1129), and CG13333 (residues 1 to 387). PCR products were cloned into pMT/V5-His-Topo (Invitrogen). CG1333 PCR product was cloned into pCR2.1 Topo (Invitrogen), and *Eco*RI-digested fragments were subcloned into pUAST-Myc vector. Vectors pMT/BiP/V5-His/GFP and pMT/lacZ (Invitrogen) were used as control for the expression of secreted and intracellular proteins, respectively. S2R+ cells (obtained from the Drosophila Genomics Resource Center, [81]) were cultured in Schneider's *Drosophila *Medium (Invitrogen) supplemented with 10% heat-inactivated fetal bovine serum and antibiotics. For transient transfections, 3 × 10^6 ^cells were transfected with 3 μg of vector DNA by using Cellfectin Reagent according to standard techniques (Invitrogen). pUAST-Myc vector was co-transfected with 4 μg of pMT/Gal4 vector (both from the Drosophila Genomics Resource Center) [81]. Expression of the constructs was induced 48 h post-transfection by adding CuSO_4 _to the cell medium (final concentration 0.5 mM). After 24 h, induced and uninduced (control) cells were harvested, transferred onto coverslips, and fixed with 4% paraformaldehyde.

### Immunostaining of S2R+ cells and embryos

Cells were fixed with 4% paraformaldehyde, permeabilized with PBS containing 0.1% saponin for 15 min, and then blocked with PBS/5% BSA/0.1% saponin for 45 min prior to incubation with primary antibodies: monoclonal anti-V5 (Sigma, 1:500) or monoclonal anti-myc (9E10, DSHB, diluted 1:20). Cells were washed three times in PBS/0.1% saponin and incubated with the secondary antibodies and probes: anti-mouse Alexa 488 (Molecular Probes, 1:500) and Alexa Fluor 546 phalloidin (Molecular Probes, 33 nM). For immunostaining of embryos, they were fixed and treated as described [40], except that 1G9 monoclonal anti-Hnt (1G9, DSHB, diluted 1:20) was used as primary antibody.

After the primary antibody, embryos were either incubated with biotinylated (Vector) or fluorochrome-associated secondary antibodies (Alexa 488; Molecular Probes 1:500 dilution), and nuclear staining was achieved with To-PRO3 (Molecular Probes, 10 μM). For biotinylated secondary antibodies, signal was revealed using the Vectastain ABC kit (Vector) according to the manufacturer's protocol, and embryos were cleared and mounted in 70% glycerol/PBS. Fluorescently labeled embryos were mounted in Dabco-Mowiol. Confocal images of cells and embryos were collected using the Confocal Laser Scanning Microscope-510 META (Zeiss, Oberkochen, Germany) and processed using LSM Image Browser software (Zeiss) and Adobe Photoshop 7.0. The pinhole diameters for each fluorescence channel were set between 1.30 μm and 1.40 μm. All images were taken using objective Plan-Apochromat 63×/1.4 Oil at 1024 pixel resolution.

### Cell fractionation

Transfected cells were collected, washed twice with PBS, once with H_2_O, and then resuspended in hypotonic buffer (50 mM Tris-HCl pH 7.5, 1 mM EDTA) plus protease inhibitors (Sigma Aldrich, St Louis, MO, USA). After 10 min in ice, cells were broken with a glass homogenizer and centrifuged at 430 × *g *for 10 min. The supernatant was recovered and centrifuged at 100,000 × *g *for 1 h in an XL-70 Beckman centrifuge using a SW 41 Ti rotor. The resulting pellet was resuspended in lysis buffer (50 mM Tris-HCl pH 7.8, 150 mM NaCl and 1% Nonidet P-40) plus protease inhibitors (Sigma); the supernatant was lyophilized and then resuspended in lysis buffer. The culture medium was recovered, lyophilized, and resuspended in SDS-PAGE sample buffer. Fractions were analyzed by SDS-PAGE and western blotting [65] with monoclonal anti-V5 (diluted 1:3000) or 9E10 monoclonal anti-myc (diluted 1:100).

### RNAi vector construction and microinjection

A 560-bp region of the third exon of CG6234 was generated by PCR using genomic DNA as template with the primers indicated in Additional file 4 (Primers used for qPCR and conventional PCR reactions). To create the knockdown plasmid UAS-*CG6234IR*, the PCR product was inserted into the pWiz vector (Drosophila Genomics Resource Center, Bloomington, IN, USA) at each of the *Avr*II and *Nhe*I restriction sites, in opposite orientations [82]. Clones were confirmed by sequencing. *w*^1118 ^embryos were injected with the UAS-*CG6234IR *construct at Genetic Services, Inc. (Sudbury, MA, USA), according to standard protocols [83]. Homozygous lines were generated with standard balancer chromosomes. RNAi experiments were repeated using three independent UAS-RNAi insertions.

## Authors' contributions

AZ participated in the design of the study and manuscript writing, designed and produced the microarrays, carried out sample preparations and microarray experiments and prepared the figures. CH carried out bioinformatic analyses, participated in sample preparation and generated and analyzed CG6234-RNAi lines. PH carried out *in situ *hybridization assays and prepared the figures. FI carried out cell transfection experiments. PM and RP carried out the sequence alignment and GO analysis. LP carried out microarray production and qPCR assays. MG participated in the design of the study and active scientific discussion. VC conceived and coordinated the study, and wrote the manuscript. All authors read and approved the final manuscript.

## Supplementary Material

Additional file 1 **Verification of SSH procedure**. **(A) **To estimate the efficiency of subtraction, the abundance of actin transcripts was analyzed by qPCR using unsubtracted (white bars) and subtracted cDNAs (red bars) from forward and reverse subtractions as templates. The result demonstrated that the abundance of actin, a non-specifically expressed housekeeping gene, was greatly decreased in subtracted samples. The unsubtracted sample is a control that is integral to the subtraction process, so it was subjected to the same dilutions and amplifications as the corresponding subtracted sample. **(B) **Differential expression of a random population of cloned genes (126 clones) was measured by filter hybridization as described in Methods. The graph shows the normalized hybridization signal intensities of each spot on the *y *axis for stages 6-7 (S6-7) and on the *x *axis for stages 2-3 (S2-3). The intensity values of 76% of the clones were at least 2-fold higher in S6-7 than in S2-3. **(C) **Virtual northern blots analysis using cDNA from syncytial blastoderm (S2-3) and gastrula (S6-7). Labeled probes corresponded to 10 clones that were four-fold overexpressed in gastrula compared to syncytial blastoderm in panel B. In each case, stronger hybridization signals were obtained with the gastrula cDNA, confirming the results from the microarray assays. Signal intensities were normalized to that of the *actin *gene. Click here for file

Additional file 2 **Functional composition of the subtracted library**. Comparison of gene ontology (GO) categories between the subtracted library and the whole *D. melanogaster *genome based on 11 Molecular Functions **(A) **and 14 Biological Processes **(B)**. The percentage (*x *axis) of each GO term (*y *axis) indicates the representation of each category within the subtracted library (red bars) or the whole genome (blue bars). Click here for file

Additional file 3 **Genes up-regulated during embryo development**. For each protein-coding gene and ncRNAs, the table displays the CG number and name, which links to FlyBase [66]. The gene ontology (GO) terms are: Molecular Function, Biological Process and Cellular Component. Homology/Protein Domain: protein domains that were identified using bioinformatics tools. Cluster number: the number of the group in which each gene was contained after the hierarchical cluster analysis. In situ Pattern: pattern of spatial expression described in our present work. Literature: a list with references citing studies in which the genes were found to be differentially expressed during *D. melanogaster *development. For intergenic regions, the table displays the clone ID, GenBank accession number, and genomic location. Click here for file

Additional file 4 **Primers used for qPCR and conventional PCR reactions**. The table contains primer names, amplicon sizes, annealing temperatures (Tm) and primer sequences. Click here for file
