# Supplementary figures and images for: Genes encoding novel secreted and transmembrane proteins are temporally and spatially regulated during Drosophila melanogaster embryogenesis
Source: BMC Biol. 2009 Sep 22;7:61. doi: 10.1186/1741-7007-7-61 (PMC2761875; doi:10.1186/1741-7007-7-61)

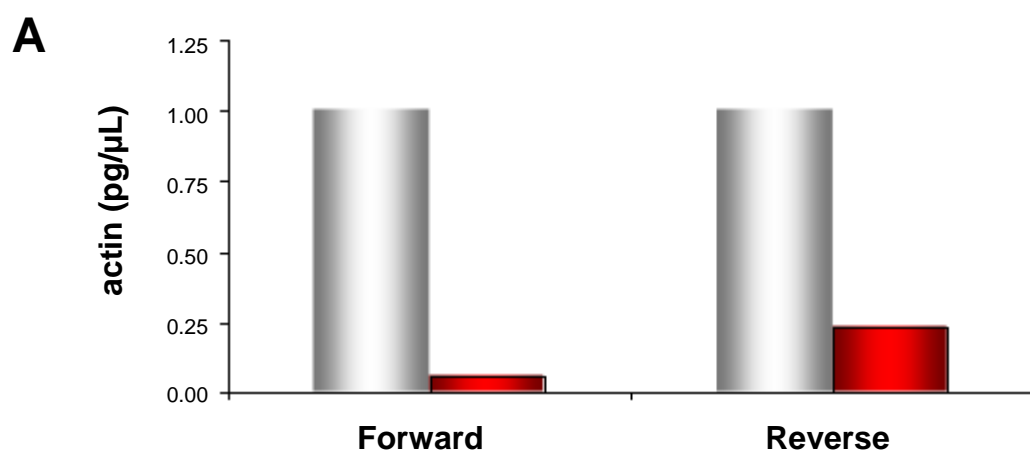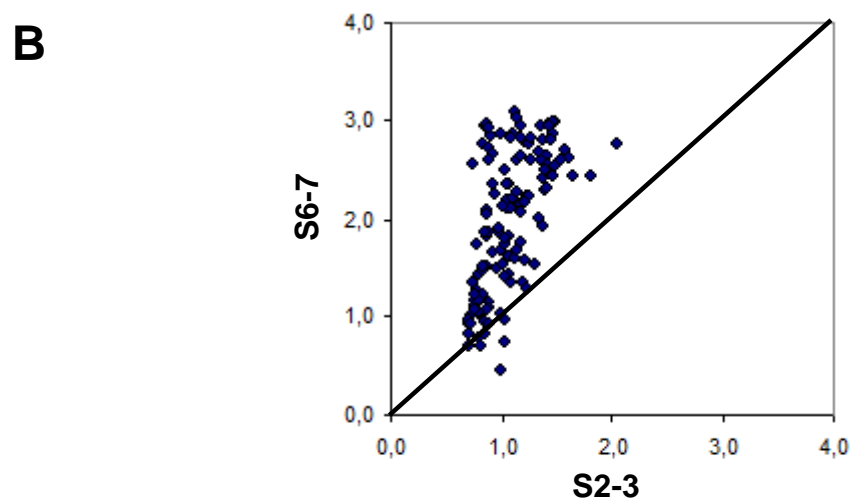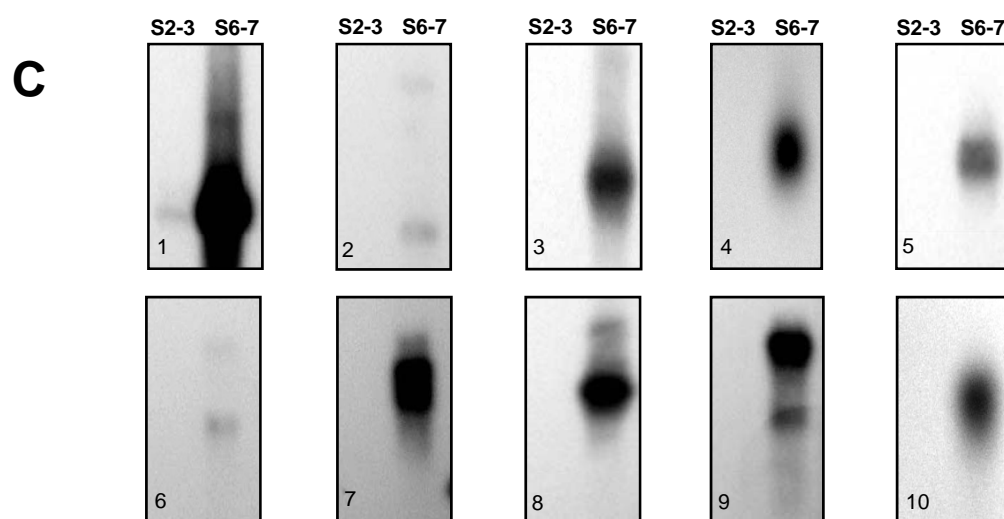

Supplement: Additional file 1 — Verification of SSH procedure. (A) To estimate the efficiency of subtraction, the abundance of actin transcripts was analyzed by qPCR using unsubtracted (white bars) and subtracted cDNAs (red bars) from forward and reverse subtractions as templates. The result demonstrated that the abundance of actin, a non-specifically expressed housekeeping gene, was greatly decreased in subtracted samples. The unsubtracted sample is a control that is integral to the subtraction process, so it was subjected to the same dilutions and amplifications as the corresponding subtracted sample. (B) Differential expression of a random population of cloned genes (126 clones) was measured by filter hybridization as described in Methods. The graph shows the normalized hybridization signal intensities of each spot on the y axis for stages 6-7 (S6-7) and on the x axis for stages 2-3 (S2-3). The intensity values of 76% of the clones were at least 2-fold higher in S6-7 than in S2-3. (C) Virtual northern blots analysis using cDNA from syncytial blastoderm (S2-3) and gastrula (S6-7). Labeled probes corresponded to 10 clones that were four-fold overexpressed in gastrula compared to syncytial blastoderm in panel B. In each case, stronger hybridization signals were obtained with the gastrula cDNA, confirming the results from the microarray assays. Signal intensities were normalized to that of the actin gene. [file 1741-7007-7-61-S1.PDF]
