# Supplementary material for: Genes encoding novel secreted and transmembrane proteins are temporally and spatially regulated during Drosophila melanogaster embryogenesis
Source: BMC Biol. 2009 Sep 22;7:61. doi: 10.1186/1741-7007-7-61 (PMC2761875; doi:10.1186/1741-7007-7-61)
Supplement: Additional file 2 — Functional composition of the subtracted library. Comparison of gene ontology (GO) categories between the subtracted library and the whole D. melanogaster genome based on 11 Molecular Functions (A) and 14 Biological Processes (B). The percentage (x axis) of each GO term (y axis) indicates the representation of each category within the subtracted library (red bars) or the whole genome (blue bars). [file 1741-7007-7-61-S2.PDF]

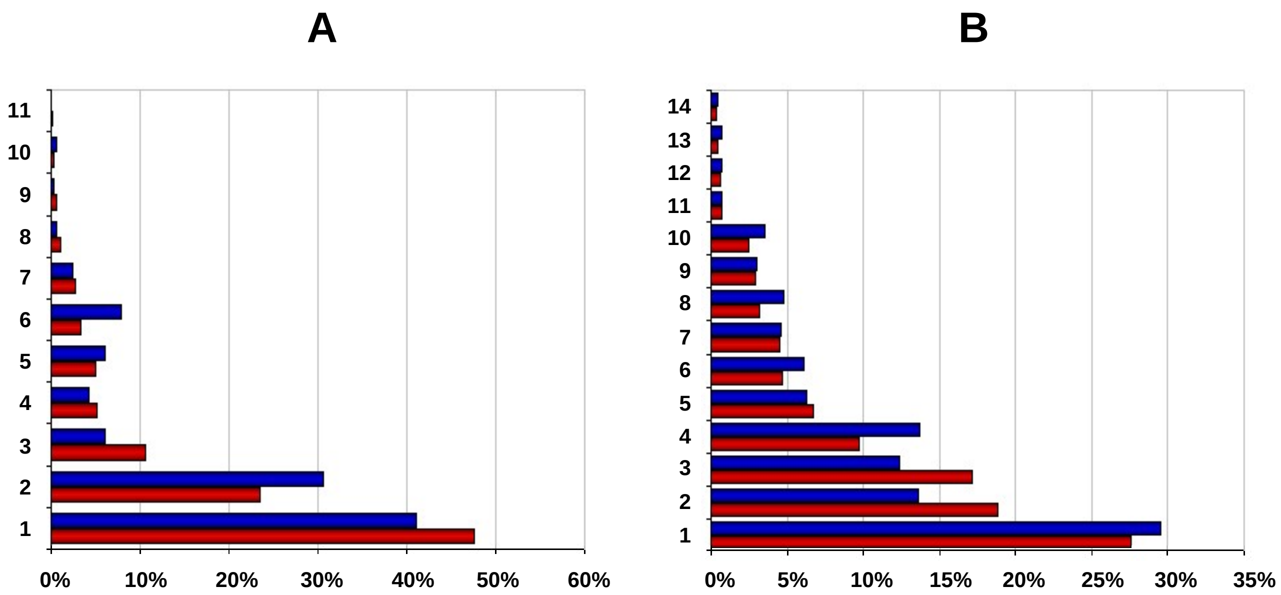

A. Molecular functions: 1, binding; 2, catalytic activity; 3, transcription regulator activity; 4, structural molecule activity; 5, molecular transducer activity; 6, transporter activity; 7, enzyme regulator activity; 8, translation regulator activity; 9, antioxidant activity; 10, motor activity; 11, auxiliary transport protein activity.

B. Biological processes : 1, cellular process; 2, developmental process; 3, multicellular organismal process; 4, metabolic process; 5, biological regulation; 6, localization; 7, gene expression; 8, establishment of localization; 9, reproduction; 10, response to stimulus; 11, biological adhesion; 12, reproductive process; 13, immune system process; 14, growth.
