# Supplementary material for: Genes encoding novel secreted and transmembrane proteins are temporally and spatially regulated during Drosophila melanogaster embryogenesis
Source: BMC Biol. 2009 Sep 22;7:61. doi: 10.1186/1741-7007-7-61 (PMC2761875; doi:10.1186/1741-7007-7-61)
Supplement: Additional file 4 — Primers used for qPCR and conventional PCR reactions. The table contains primer names, amplicon sizes, annealing temperatures (Tm) and primer sequences. [file 1741-7007-7-61-S4.DOC]

**Primers used for the real-time quantitative PCR and standard PCR reactions.**

| **Primer name** | **Length (bp)** | **Tm** | **Sequence (5`→ 3`)** |
| --- | --- | --- | --- |
| *Actin-s* | 280 | 60 | CACCGGTATCGTTCTGGACT |
| *Actin-as* |  |  | CTCGTAGGACTTCTCCAACG |
| *DmCG1225-s* | 753 | 72 | GGAGTCTCTCGGGACACTACATC |
| *DmCG1225- as* |  |  | CGGTTGTACTTCGTCTTACGGCG |
| *DmCG11212-s* | 132 | 66 | GAGCTGGGCGTTGCAGGATATA |
| *DmCG11212-as* |  |  | CGAGGACATGTATGTGTAGCAG |
| *DmCG6234-s* | 213 | 62 | GCCAATCGAGTCAACCTCATT |
| *DmCG6234-as* |  |  | CCGCCGTAAACCGATACAAAA |
| *DmCG17957-s* | 425 | 52 | GCTTTGACGGCTTTGTG |
| *DmCG17957-as* |  |  | TGTGCGGATGAAGTGGA |
| *IR-CG6234-s* | 560 | 62 | TCTAGAACATTGCCGTTTCCAGTGAGC |
| *IR-CG6234-as* |  |  | TCTAGACACCACAGTTGCGTCTACAG |
